# A First-Principles Study of Nonlinear Elastic Behavior and Anisotropic Electronic Properties of Two-Dimensional HfS_2_

**DOI:** 10.3390/nano10030446

**Published:** 2020-03-01

**Authors:** Mahdi Faghihnasiri, Aidin Ahmadi, Samaneh Alvankar Golpayegan, Saeideh Garosi Sharifabadi, Ali Ramazani

**Affiliations:** 1 Computational Materials Science Laboratory, Nano Research and Training Center, Tehran 19967-15433, Iran; mahdi.faghihnasiri@gmail.com (M.F.); aidin.ahmadiii@gmail.com (A.A.); 2 Department of Physics, K.N. Toosi University of Technology, Tehran 19967-15433, Iran; sama.alvankr@gmail.com (S.A.G.); sgarosi@mail.kntu.ac.ir (S.G.S.); 3 Department of Mechanical Engineering, Massachusetts Institute of Technology, 77 Massachusetts Ave., Cambridge, 02139 MA, USA

**Keywords:** HfS_2_, density functional theory, mechanical properties, bandgap, elastic constants

## Abstract

We utilize first principles calculations to investigate the mechanical properties and strain-dependent electronic band structure of the hexagonal phase of two dimensional (2D) HfS_2_. We apply three different deformation modes within −10% to 30% range of two uniaxial (D1, D2) and one biaxial (D3) strains along *x*, *y*, and *x*-*y* directions, respectively. The harmonic regions are identified in each deformation mode. The ultimate stress for D1, D2, and D3 deformations is obtained as 0.037, 0.038 and 0.044 (eV/Ang3), respectively. Additionally, the ultimate strain for D1, D2, and D3 deformation is obtained as 17.2, 17.51, and 21.17 (eV/Ang3), respectively. In the next step, we determine the second-, third-, and fourth-order elastic constants and the electronic properties of both unstrained and strained HfS_2_ monolayers are investigated. Our findings reveal that the unstrained HfS_2_ monolayer is a semiconductor with an indirect bandgap of 1.12 eV. We then tune the bandgap of HfS_2_ with strain engineering. Our findings reveal how to tune and control the electronic properties of HfS_2_ monolayer with strain engineering, and make it a potential candidate for a wide range of applications including photovoltaics, electronics and optoelectronics.

## 1. Introduction

The rise of two-dimensional (2D) materials began in 2004 with a focus on graphene sheets by Novoselov and Geim [1]. Graphene is a 2D layer of sp^2^-bonded carbons as the first prototype of 2D layered materials, which is viewed as an ideal material for a wide range of applications including photonics, THz electronics, nonlinear optics, sensors, and transparent electrodes [2,3,4,5]. 2D materials have been intensively researched for the next generation of ultrathin and flexible electronic and optoelectronic devices, including transistors, phototransistors, solar cells, and light-emitting diodes (LEDs) [6,7,8,9]. These materials have historically been one of the most extensively studied classes of materials due to their wealth of significant physical phenomena, which can occur when charge and heat transports are confined to a 2D surface [10,11,12].

Recently, atomically thin 2D materials, such as graphene, hexagonal boron nitride (*h*-BN), and the transition-metal dichalcogenides (TMDs) have received a lot of interests due to their unique electronic and optoelectronic properties. The TMDs with a general formula of MX_2_ (M = transition metal, X = S, Se, Te) are particularly an interesting class of 2D materials comprising both metalic and semiconducting behaviors [10]. Semiconducting TMDs have advantages over gapless graphene in their application for logic transistors since their sizable bandgap is necessary to achieve high on/off ratio [12]. Among TMDs, MoS_2_ is the most widely investigated as a semiconducting TMD. MoS_2_-based transistors have shown an extremely high room-temperature current on/off ratio of ≈ 108 and mobility of higher than 200 cm^2^/(V s), which is comparable to the mobility achieved in thin silicon films [13] and graphene nanoribbons [14,15,16,17]. Other members in the TMD family are still in the stage of exploration. For example, group IVB (Hf and Zr)-based TMDs are theoretically predicted to have higher mobility (mobility of HfS_2_ = 1833 cm^2^/(V s), HfSe_2_ = 3579 cm^2^/(V s), ZrS_2_ = 1247 cm^2^/(V s), ZrSe_2_ = 2316 cm^2^/(V s)) and higher sheet current density than group VIB (Mo and W)-based TMDs [6,18,19]. In contrast to graphene with zero bandgap, TMD-VIB monolayers possess sizable electrical performance [3,4,20,21] and optoelectronic properties [6,22,23]. For instance, the HfS_2_ monolayer is semiconductor with an indirect bandgap of about 2eV, according to the experimental measurements [18,19,24,25].

In addition, ultrathin HfS_2_ not only shows faster and higher response, but also higher stability in comparison to most of the other 2D materials, which makes HfS_2_ an appropriate positional candidate for the electronic and optoelectronic applications [7,26].

The HfS_2_ monolayer shows isotropic elastic parameters (i.e. in-plane stiffness and Poisson’s ratio), which are the same as those for MoS_2_ in both armchair and zigzag directions. When the strain along both x and y directions increases, the bandgap of HfS_2_ increases, while the bandgap of MoS_2_ decreases. Therefore, the same as MoS_2_, the band structure of HfS_2_ can also be effectively tuned by applying uniaxial strains [27].

In the current research, we first employ density functional theory (DFT) to study the mechanical properties (strain-stress energy), and elastic constants under different deformation modes. Then, we do strain engineering to tune the electronic properties of the HfS_2_ monolayer and make it a potential candidate for different applications.

## 2. Computational Details

We performed DFT calculations with the Quantum ESPRESSO package [28,29] using projector-augmented wave (PAW) method. We used the Perdew–Burke–Ernzerhof exchange-correlation functional, revised for solids (PBEsol) along with the projector-augmented wave (PAW) potentials for the selfconsistent total energy calculations and geometry optimization [30]. Eighteen valence electrons of Hf atoms (4f^14^ 5d^2^ 6s^2^) and six valence electrons of S atoms (3s^2^ 4p^4^) were included in the computations. For the plane-wave expansion, the cutoff energy after convergence is set to 880 eV. The Brillouin zone sampled using a 20 × 20 × 1 Monkhorst–Pack *k*-point grid [31]. Atomic positions were relaxed until the energy differences are converged within 10^−6^ eV and the maximum Hellmann– Feynman force on any atom is below 10^−6^ eV. A vacuum of 15 Å along the c direction was included to safely avoid the interaction between the periodically repeated structures. Under various deformation tensors, the total energy of the system is calculated and led to achieve energy-strain curves. Here, the strained energy per atom is defined as below:

(1) $$\;E_{S}=\frac{E_{tot}-E_{0}}{n}$$

Where *E*_tot_, and *E*_0_ are the total energy of the strained and unstrained HfS_2_ monolayers, respectively. *n* is also the number of atoms in the unit cell. The DFT simulation calculates the true or Cauchy stresses, σ, which for the HfS_2_ monolayer should be expressed as a 2D force per length with the unit of N/m by taking the product of the Cauchy stresses (with the unit of N/m^2^) and the super-cell thickness of 15 Å. The Cauchy stresses are related to the second Piola–Kirchhoff (PK2) stresses Σ as [32]:

(2) $$\Sigma =JF^{-1}\sigma {\left(F^{-1}\right)}^{T}$$

where, *F* is the deformation gradient tensor [32], J is the determinant of *F*, and σ is the true stress with the unit of *N*/m. In continuum theory of elasticity [32], and finite element method [33], the second *P*-*K* stress is employed to explore the impact of large forces on the mechanical behavior of materials [34]. Polynomial fitting of the resultant second *P*-*K* strain-stress curves on the DFT results has been conducted to calculate the second-, third-, and fourth-order elastic constants using continuum theory of elasticity. 

To identify the elastic constants, the obtained strain-stress curves from DFT calculations are fitted to the constitutive equations of the continuum theory of elasticity. The second-order elastic constants (C_11_, C_12_, C_12_, and C_66_) are the representative of the linear elastic response of the structure, while the higher-order (third-, and fourth-s order) constants are essential to the study of nonlinear elastic behavior of materials, as Wei et al. [35], Peng et al. [32], and Faghihnasiri et al. [6,7,8,9] described it completely for Boron nitride, graphene, and borophene monolayers, respectively. To identify the elastic constants, suitable deformations should be selected to facilitate the calculation of these constants directly from the stress-strain curves. For this purpose, three different types of deformations modes (strain tensors (D_1_, D_2_, and D_3_) ) is this study, which are previously defined by Wei et al. [35], Peng et al. [32], and Faghihnasiri et al. [9].

Additionally, using the second-order elastic constants, bulk modulus (*K*), shear modulus (*G*), Young’s modulus (*Y*) and Poisson’s ratio (*ν*) can calculated in *x* and *y* directions. We utilize the following formulas for 2D materials to calculate these parameters [36,37]:

(3) Yx2D=(C11 C22−C122)C11, Yy2D=(C11 C22−C122)C22

(4) $$v_{x}^{2D}=C_{12}/C_{11},\;v_{y}^{2D}=C_{12}/C_{22}$$

(5) $$G^{2D}=C_{66}$$

(6) $$K_{x,y}=\frac{Y_{x,y}^{2D}}{2\left(1-v_{x,y}^{2D}\right)}$$

## 3. Results and Discussion

### 3.1. Atomic Structure

**Figure 1:**
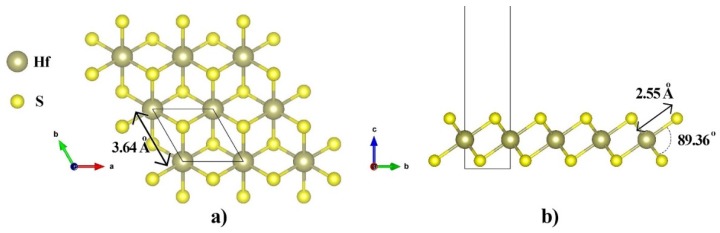
(**a**) Top view and (**b**) side view of the atomic structure of the hexagonal HfS_2_ monolayer. The light brown and yellow balls represent Hf and S atoms, respectively. The black rectangle denotes the unit cell.

HfS_2_ monolayer is in a hexagonal phase with an inversion center at the Hf atom sites. Each Hf atom is bounded to six S atoms. The unit cell consists of one Hf atom, and two S atoms. The geometric structure of a HfS_2_ monolayer is depicted in Figure 1. The obtained optimized lattice constant of HfS_2_ is 3.64 Å, which is in good agreement with the lattice constant of bulk HfS_2_, which is reported as 3.63 Å using PBE based calculations [38], and 3.61 Å utilizing vdW-TS/HI method [39]. As can be seen in Table 1, the optimized lattice constant of HfS_2_ is in a geed agreement with reported lattice constant of similar 2D materials including HfSe_2_, ZrS_2_, ZrSe_2_, GaS, GaSe, and InSe in the literature. The bond length between Hf and S atoms is also calculated as 2.55Å (Figure 1b). This value is in good agreement with reported data in the literature (2.59 Å) [40]. The bond angle between Hf and S atoms (atoms No. 1, 2, and 3) is 88.80°.

### 3.2. Mechanical Properties

**Figure 2:**
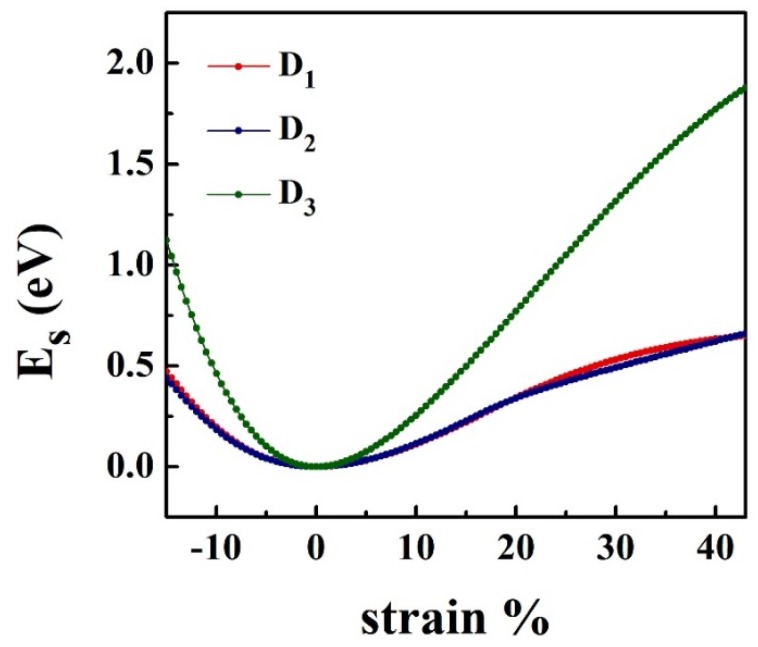
The energy-strain per atom of HfS_2_ monolayer under the uniaxial strain along *x* (D_1_) and *y* (D_2_) directions, and biaxial strain along *x*-*y* (D_3_).

The energy-strain curves for HfS_2_ monolayer under three types of deformations, namely uniaxial strain along *x* (D_1_), *y* (D_2_) and biaxial strain along *x*-*y* (D_3_), is analyzed and demonstrated in Figure 2. It is evident that $E_{s}$ differs from the applied strain along with *x* and *y* directions. For the tensile and compressive strains through all three modes, $E_{s}$ becomes asymmetric. The strained energy is a quadratic function of strain in the range between −3%≤η≤5% for the uniaxial strain along *x* direction. For the uniaxial strain along y direction and biaxial strain, these harmonic region ranges between −7%≤η≤3% and −2%≤η≤2%, respectively. 

In all three deformation modes, the total energy of the system increases with increasing the applying strain. As can be seen in Figure 2, The changes of *E*_s_ with strain for D_1_ and D_2_ deformation modes is almost the same. However, for D_3_ deformation mode, the rate of energy change with strain is much higher (Figure 2).

### 3.3. Strain-Stress Relationship

**Figure 3:**
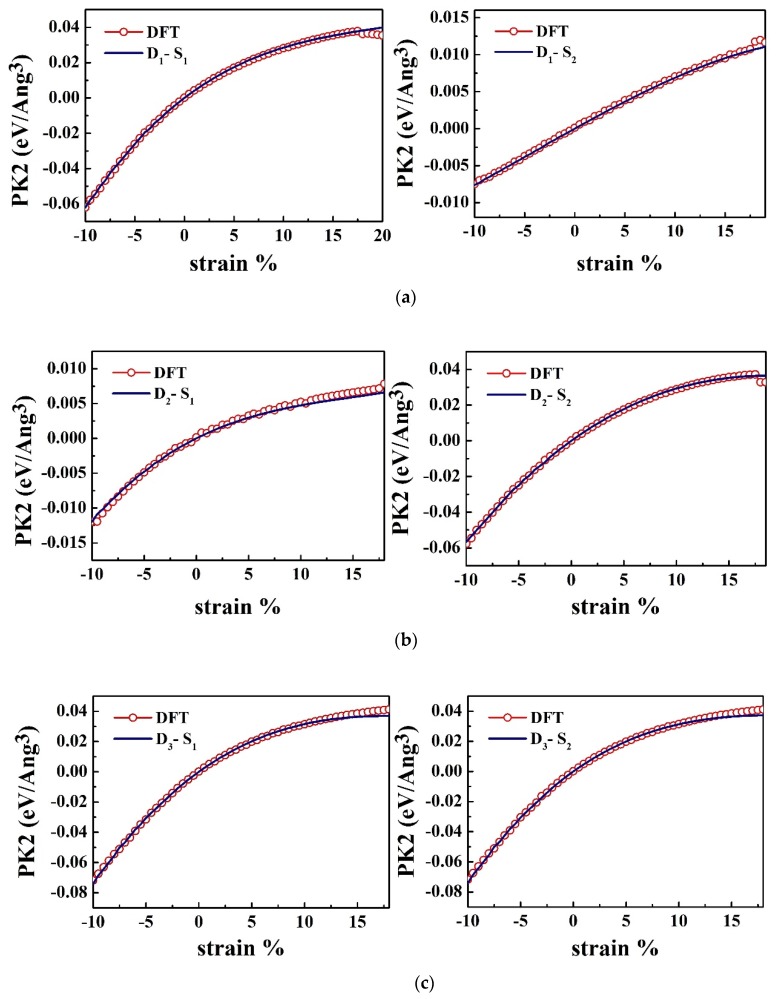
Strain-stress relationships for three types of strain, namely (**a**) D_1_ (**b**) D_2_ (**c**) D_3_, fitted with *S*_1_ and *S*_2_, which denote to the *x* and *y* components of the stress, respectively.

Figure 3 shows the strain-stress relations obtained from the DFT calculations as well as the fits to these by the equations of continuum theory of elasticity. As can be seen in this figure, the stress-strain curves are depicted for all D_1_, D_2_, and D_3_ deformation modes. 

**Figure 4:**
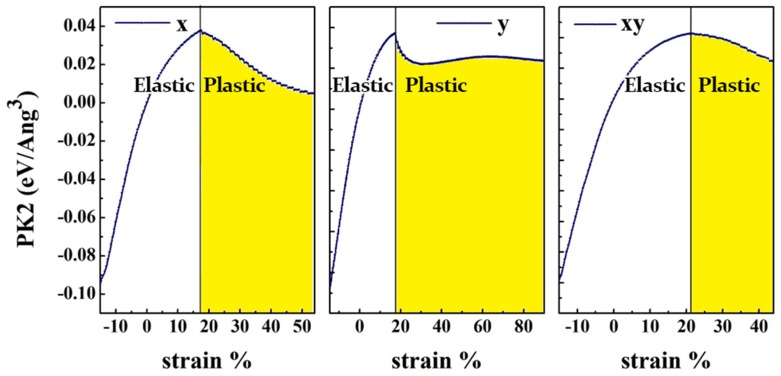
Σ*_m_* and *η_m_* denote the *x*, *y*, and *x*-*y* components of the stress and strain, respectively. The line separates the harmonic and anharmonic regions. The yellow area shows the plastic region.

The maximum value in the stress-strain curves shows the ultimate tensile strength (Σ*_m_*) of the material, in which a material can suffer the maximum respective *η_m_* without damaging (Figure 4). The ultimate strain reflects the intrinsic bonding strengths and acts as a lower limit of the critical strain. Additionally, the values obtained for the ultimate stress and ultimate strain is given in Table 2. Beyond the ultimate strain, the materials will get in a metastable state, which ends up with fracture [42]. The DFT results for strains below the ultimate strain are used to determine the higher-order elastic constants.

As can be seen in Figure 4, the stress increases linearly with increasing strain, within the harmonic (elastic) region. Under larger strains, for the prediction of strain-stress curves, the system is in the anharmonic region in which higher-order terms must be perceived as well. As mentioned previously, the system transits from elastic to the plastic region, when exposed to higher strains. Eventually, Table 3 prepares the nonzero second-, third- and fourth- order elastic constants (SOEC, TOEC and FOEC, respectively) for the HfS_2_ monolayer.

To comprehensively understand the magnitudes of elastic constants obtained in this work for HfS_2_, Table 4 presents multiple comparisons between our findings and the other reported elastic constants for similar structures. Furthermore, 2D Young’s moduli (in-plane stiffness) along *x* and *y* directions ($Y_{x}^{2D}$, $Y_{y}^{2D}$), Poisson’s ratio along *x* and *y* directions ($v_{x}^{2D}$, $v_{y}^{2D}$), 2D shear modulus (*G*^2D^), and the 2D bulk modulus (*K*), are tabulated in Table 5 and validated by those reported for other structurally similar compounds in the literature. 

### 3.4. Electronic Properties

**Figure 5:**
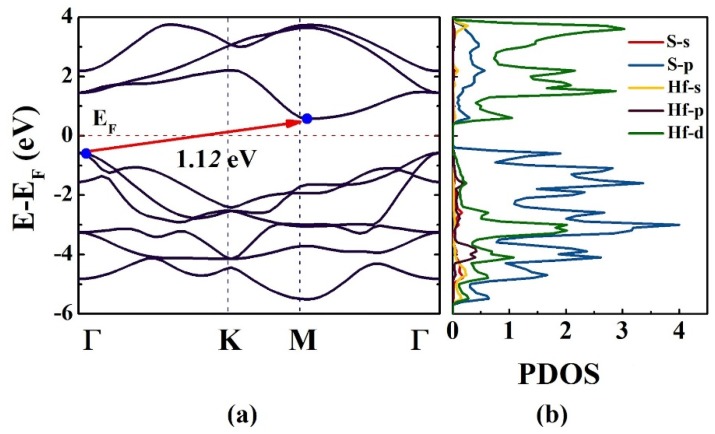
Band structure (**a**) and PDOS (**b**) of the HfS_2_ monolayer in free-strain condition. The Fermi level is set to zero.

First, we studied the electronic properties of HfS_2_ in the absence of strain. Figure 5 shows the band structure of the strained structure of HfS_2_, which is obtained from PBEsol calculations. As can be seen in Figure 5, HfS_2_ is a semiconductor with an indirect bandgap of 1.12 eV. This value is compared with other methods of HfS_2_ ($E_{g}^{LDA}$= 1.07 eV [40], $E_{g}^{GGA}$= 1.15 eV [43], $E_{g}^{\mathrm{HSE}06}$= 2.02 eV [44], and $E_{g}^{GW}$= 2.45 eV [45]), where it is in a good agreement with $E_{g}^{GGA}$= 1.15 eV [43]. As can be seen in Table 4, The predicted gap energies by GW and HSE06 are larger than the predicted gap energy by PBE approximation. Since the HSE06 functional can accurately predict enthalpies of formation, ionization potentials, and electron affinities for lattice constants and band gaps of solids in general, the predicted gap energy by HSE06 is larger and more accurate than the predicted gap energy by PBE [46]. Also, in GW calculations, the excitonic effects are included and due to the fact that the excitonic effects are significant in 2D semiconductors, the predicted gap energy by GW is twice larger than the predicted gap energy by GGA approximation for the HfS_2_ monolayer (Table 4) [47].

In our predicted electronic structure (Figure 5), it can be seen that the conduction-band minimum (CBM) and valence-band maximum (VBM) are located at M and Γ points, respectively. Additionally, we examine the projected density of states (PDOS), which demonstrates the contribution of orbitals in the valence and conduction bands of the material. In the conduction band, Hf-5d orbitals have the maximum contribution. In contrast, the 3p orbital of S atom makes a greater contribution in the valance band near the Fermi level, as shown in Figure 5b.

**Figure 6:**
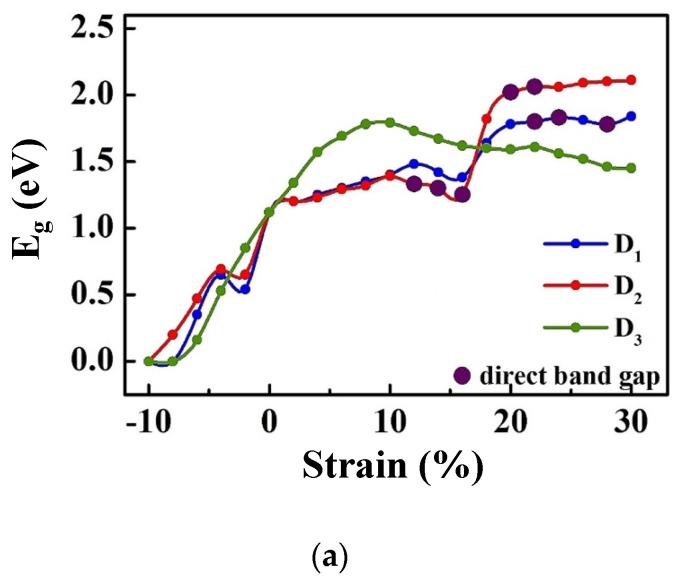

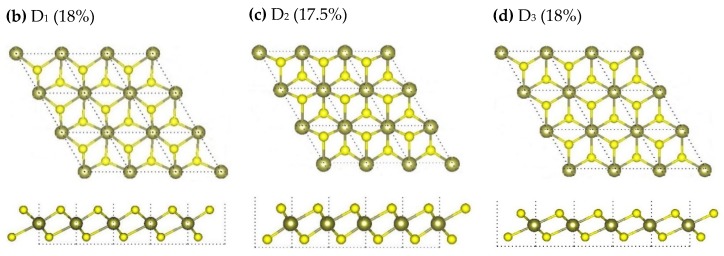
(**a**) The bandgap variations of the HfS_2_ monolayer under uniaxial strain along *x* (D_1_) and y (D_2_) directions and the biaxial strains along *x*-*y* (D_3_). (**b**) The deformed lattice structures of the HfS_2_ monolayer at uniaxial 18% tensile strain along *x* direction (D1), (**c**) the deformed lattice structures of the HfS_2_ monolayer at under uniaxial strains of 17.5% along y direction, and (**d**) the deformed lattice structures of the HfS_2_ monolayer at 18% biaxial strain along *x*-*y* directions (D3). The top and side views are shown in the top and bottom panels, respectively.

In the next step, we investigated the electronic behavior of HfS_2_ under different strains. The bandgap variation of HfS_2_ monolayer under these three types of strain are shown in Figure 6a. as can be seen in this figure, for all deformation modes, the bandgap decreases when the compressive strain increases 0% to 10%, while it increases when the tensile strain increases from 0% to 10%. In this strain range, we note that the rate of the gap energy decrease/increase with strain is almost the same for D1 and D2 deformation modes. From 10% to 16% tensile strain, the energy gap decreases for all deformation modes. For the strain range from 16% to 20%, the energy gap increases by increasing strain for D1 and D2 deformations, while it keeps decreasing for D3. For the tensile strain ranging from 20% to 30%, the gap energy is almost constant with strain for D1 and D2, while it keeps decreasing for D3. At 22%, 24%, 28% strains along x direction (D_1_), and at 12%, 14%, 16%, 20%, 22% strains along *y* direction (D_2_), the bandgap becomes direct. Under biaxial strain (D_3_), the semiconducting monolayer maintains its indirect nature, while its energy gap increases with increasing strain and reaches it maximum value (1.79 eV) at 10% strain. For the uniaxial strain in both D_1_ and D_2_ cases, when the compressive strain increases from zero to 2%, the energy gap decreases and then gradually increases by increasing the amount of the compressive strain from 2% to 5%. Then, by increasing the strain from 5% to 10%, the energy gap reduces again. For D3 case, the energy gap decreases by increasing the compressive strain from 0 to 10% deformation. As can be seen in Figure 6a, the material becomes metal (*E_g_* = 0 eV) at 10% compressive strain in all three deformation modes. The value of energy gap is the same (1.6 eV) for D_1_ and D_3_ at 18% strain, and for D_2_ at 17.5% strain. As can be observed in Figure 4, at 18% D1 and 17.5% D2 types of deformation, the material is in plastic region, while at 18% D_3_ type deformation, the material shows elastic deformation (Figure 6b,c,d). As can be seen in Figure 6d, the elastic to plastic transition for D3 occurs at 22% strain. 

**Figure 7:**
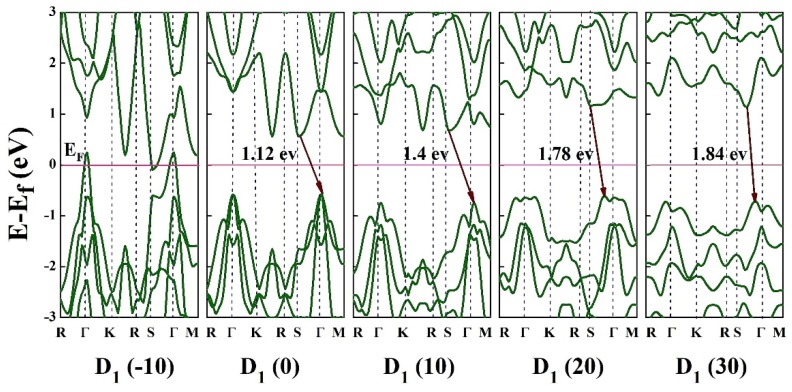
Band structure of HfS_2_ monolayer under the uniaxial strains in the range of −10% to 30% along x direction. The Fermi level is set to zero.

Our findings indicate that the electronic properties of HfS_2_ can be effectively tuned by applying planar forces to HfS_2_ in different directions. Figure 7 shows the band structures of the HfS_2_ monolayer under uniaxial compressive and tensile strains along the *x* direction (D_1_) within the range of −10% to 30%. As can be seen in Figure 7, when the strain ranges from −10% to −8%, bands of energies cross the Fermi level so that HfS_2_ at these strains under D_1_ deformation shows a metallic behavior. By reducing the value of strain to −6%, the HfS_2_ monolayer becomes an indirect semiconductor with a bandgap of 0.35 eV. At the strains above 22%, the bandgap becomes direct and energy of bandgap increases up to 1.8 eV for the D_1_ case.

In Figure 7, the conduction-band minimum (CBM) is located at *S* point, while the valence-band maximum (VBM) is located at the *Γ* point, which is shifted to the *S* point by increasing the strain. These points indicate the transformation of bandgap from indirect to direct with strain engineering.

**Figure 8:**
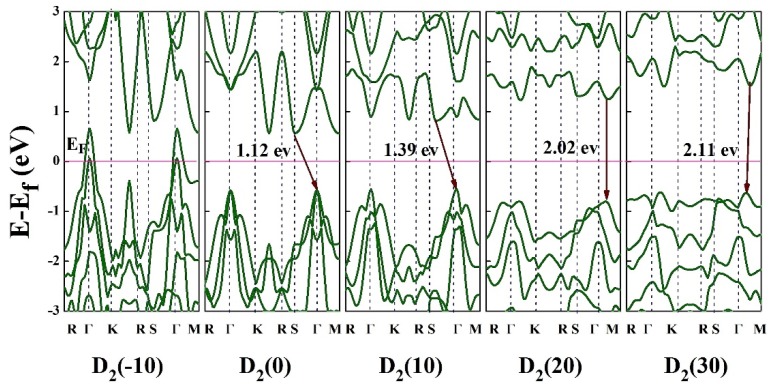
Electronic band structure of HfS_2_ monolayer under the uniaxial strains in the range of −10% to 30% along *y* direction. The Fermis level is set to zero.

For the deformed HfS_2_ under the tensile strain along *y* direction (D_2_), by increasing the strains from 0% to 30%, the bandgap increases from 1.12 eV to its maximum value of 2.11 eV continuously. The band structure of strain HfS_2_ monolayer under uniaxial compressive and tensile strain along *y* direction (D_2_) is shown in Figure 8. Under the compressive strain of D_2_ deformation, there is no bandgap near the Fermi level so the system is metallic. In addition, it remains as an indirect semiconductor over the entire applied compressive strain domain when it is strained along *y* direction (D_2_). However, under tensile straining, at 12% to 22% strains, the gap is direct and energy bandgap changes from 1.33 to 2.06 eV. When the compressive strain is applied (D2), the located CBM at *S* point moves to *Γ* point, and the VBM at the *Γ* point moves to *M* point. By applying tensile strain, CBM gets away from the Fermi level and the bandgap increases from 1.12 eV (at unstrained condition) to 2.11 eV (strained with 30% tensile strain).

**Figure 9:**
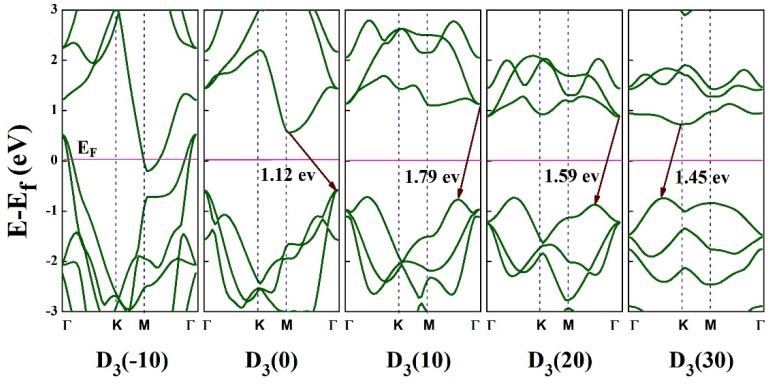
Band structure of the HfS_2_ monolayer under D_3_ deformation in the range of −10% to 30%.

In Figure 9 the band structure of strained HfS_2_ monolayer under biaxial strain along *x*-*y* (D_3_) ranging from −10% to 30% strain is shown. As can be seen in Figure 9, under compressive strain ranging from −10% to −6% strains, the bands cross the Fermi level and the system shows metallic behavior. At −6% strain and beyond (−6 < strain < 0), the HfS_2_ monolayer becomes an indirect semiconductor with a bandgap of 0.16 eV in −6% strain. In the tensile deformation domain, the gap energy increases increasing the stain. At 0%, 10%, 20%, and 30% strains, the bandgap becomes 1.12 eV, 1.79 eV, 1.59 eV and 1.45 eV, respectively. The CBM is located at *M*, *Г, Г* and *K* point for 0%, 10%, 20%, and 30% strain, respectively. Also, the VBM at 0%, 10%, 20% strains are located between M and *Г* points and at 30% strain, it is located between *Г* and *K* points.

**Figure 10:**
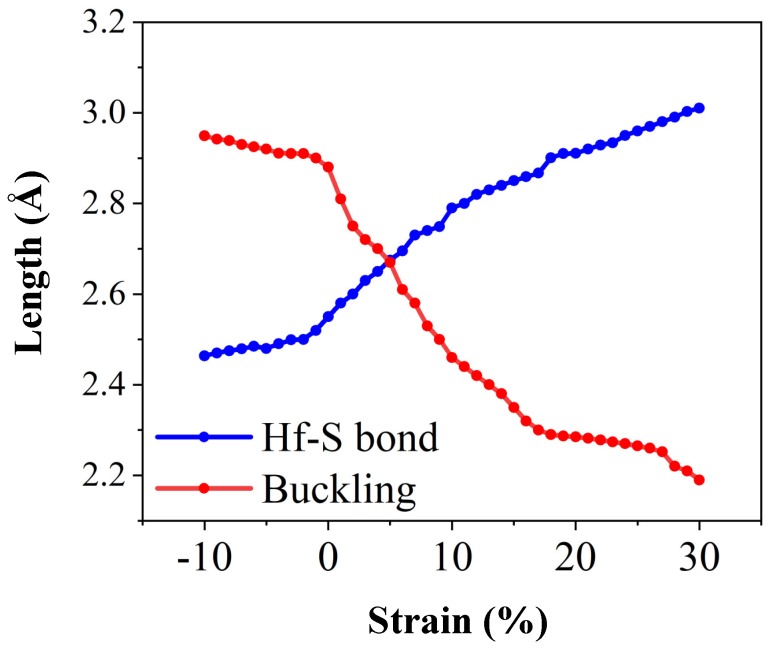
Hf-S bond length and buckling of the HfS_2_ monolayer under D_3_ deformation in the range of −10% to 30% straining.

Graphene has a plate structure and its symmetry is not broken during deformation. Therefore, not only graphene does not suffer any bulking during deformation, but also only monotonic changes can occur on its electron properties during the biaxial straining (Figure 10) [48]. While, the HfS_2_ structure is not a plate like structure, its electronic properties are affected by the occurred buckling during the deformation. It is expected to observe many changes in the symmetry of the structure and the distance and angle of the atoms in the strained HfS_2_ monolayers. Such structural changes can greatly affect the electronic properties and therefore, the bandgap changes of HFS_2_ will not be monotonic under biaxial strains (Figure 6).

## 4. Conclusions

In summary, the mechanical and electronic properties of the HfS_2_ monolayer under two uniaxial (D_1_ and D_2_) and one biaxial (D_3_) DFT calculations is investigated. We determined the harmonic regions in the different deformation modes. This harmonic region ranges in −3%≤η≤5%, −7%≤η≤3% and −2%≤η≤2% for D_1_, D_2_, and D_3_, respectively. Our findings reveal that the ultimate stress of the HfS_2_ monolayer for D_1_, D_2_, and D_3_ is $0.037\;\frac{eV}{{Å}^{3}}\;$, $0.038\;\frac{eV}{{Å}^{3}}$,and $0.044\;\frac{eV}{{Å}^{3}}$, respectively. The obtained ultimate strain is 17%, 17.5% and 21% strain for D_1_, D_2_, and D_3_ respectively. The high order of elastic constants including second-, third-, and fourth-order constants are calculated. The values of 2D Young’s moduli along *x* and *y* directions are predicted as 83.01 N/m and 83.57 N/m, respectively. The value of Poisson’s ratio along *x* and *y* directions is the same (0.17) for both D1 and D2.

Moreover, the electronic properties of HfS_2_ show that it is a semiconductor with an indirect bandgap of 1.12 eV. The projected density of states (PDOS) indicates the conduction band, Hf-5d orbital possesses the maximum contribution, while the 3p orbital of S atom have greatest contribution in the valance band. The variation of band structure and bandgap of the HfS_2_ monolayer under D_1_, D_2_, and D_3_ deformation modes in the range of −10% to 30% are also investigated. We tuned the bandgap state (direct vs. indirect), gap energy (opening vs. shrinking), and phase transition (semiconductor- metal) by strain engineering under different deformation modes. Our findings reveal how to utilize strain engineering to make HfS_2_ monolayer as a suitable candidate for a wide range of applications including flexible solar cells, electronics and optoelectronics. 

## Figures and Tables

**Table 1:** Comparison of the lattice constants of 2D HfS_2_ are calculated using different exchange-correlation functions along with the other reported values in the literature.

Material	Method	Lattice Constant
HfS_2_	PBEsol (This work)	3.64
	GGA [40]	3.54
	HSE [40]	3.53
	LDA [40]	3.38
	PBE (bulk) [41]	3.54
	PBE (bulk) [38]	3.63
	vdW-TS/HI [39]	3.61
HfSe_2_	vdW-TS/HI [39]	3.70
ZrS_2_	vdW-TS/HI [39]	3.64
ZrSe_2_	vdW-TS/HI [39]	3.74
GaS	DFT-PBE [41]	3.64
GaSe	DFT-PBE [41]	3.82
InSe	DFT-PBE [41]	4.09

**Table 2:** Ultimate strains (*η_m_*) and ultimate stresses (Σ*_m_*) for three (D_1_, D_2_, and D_3_) types of strains.

	Uniaxial (*x*)	Uniaxial (*y*)	Biaxial (*x*-*y*)
$\Sigma_{m}$(eV/Å3) $\eta_{m}$	0.037 17.2%	0.038 17.51%	0.044 21.17%

**Table 3:** Nonzero second-, third- and fourth-order elastic constants (in N/m) for the HfS_2_ monolayer.

SOEC		TOEC		FOEC	
C_11_	86.29	C_111_	−683.81	C_1111_	3389.08
C_12_	15.28	C_112_	−14.69	C_1112_	−343.81
C_22_	85.71	C_222_	−561.10	C_2222_	1092.89
C_66_	68.14	C_122_	−145.42	C_1222_	968.49
		C_166_	−205.85	C_6666_	−592.82
		C_266_	−1118	C_1266_	2386.80
				C_1122_	−42.83
				C_2266_	−2207.16
				C_1166_	471.75

**Table 4:** Elastic constants C_11_ and C_12_ obtained in this work along with those reported in a previous works (in units of N/m).

Material	C_11_	C_12_
HfS_2_ (This work)	86.29	15.28
GaS [41]	83	18
GaSe [41]	70	16
InSe [41]	51	12
h-BN [32]	293.2	66.1
ZrS_2_ [39]	131.47	25.63
ZrSe_2_ [39]	104.62	21.31
HfS_2_ [39]	141.98	25.95
HfSe_2_ [39]	116.88	22.30

Ref [39] is in the bulk structure.

**Table 5:** 2D Young’s moduli, Poisson’s ratio, 2D shear modulus, and 2D bulk modulus for some 2D materials.

Material	$Y_{x}^{2D}\;(N/m)$	$Y_{y}^{2D}\;(N/m)$	$v_{x}^{2D}$	$v_{y}^{2D}$	$G^{2D}\;(N/m)$	K (N/m)
HfS2	83.01	83.57	0.17	0.17	68.14	50.85
ZrS2 [39]	57.22		0.20		23.85 (GPa)	31.73 (GPa)
ZrSe2 [39]	52.33		0.19		22.02 (GPa)	27.99 (GPa)
HfSe2 [39]	69.59		0.19		29.34 (GPa)	36.87 (GPa)
h-BN [32]	279.2		0.2176		-	160 (GPa)
